# Probiotic Supplementation Improves Cognitive Function and Mood with Changes in Gut Microbiota in Community-Dwelling Older Adults: A Randomized, Double-Blind, Placebo-Controlled, Multicenter Trial

**DOI:** 10.1093/gerona/glaa090

**Published:** 2020-04-17

**Authors:** Chong-Su Kim, Jiah Cha, Minju Sim, Sungwoong Jung, Woo Young Chun, Hyun Wook Baik, Dong-Mi Shin

**Affiliations:** 1 Department of Food and Nutrition, College of Human Ecology, Seoul National University, Republic of Korea; 2 Seoul W Internal Medicine Clinic, Republic of Korea; 3 Department of Psychology, Chungnam National University, Daejeon, Republic of Korea; 4 Department of Internal Medicine, Clinical Nutrition and Metabolism, Bundang Jesaeng Hospital, Seongnam, Republic of Korea; 5 Research Institution of Human Ecology, Seoul National University, Republic of Korea

**Keywords:** Probiotics, RCT, Gut microbiota, Cognitive function, Mood status, Healthy older adults

## Abstract

Probiotics have been proposed to ameliorate cognitive impairment and depressive disorder via the gut–brain axis in patients and experimental animal models. However, the beneficial role of probiotics in brain functions of healthy older adults remains unclear. Therefore, a randomized, double-blind, and placebo-controlled multicenter trial was conducted to determine the effects of probiotics on cognition and mood in community-dwelling older adults. Sixty-three healthy elders (≥65 years) consumed either placebo or probiotics containing *Bifidobacterium bifidum* BGN4 and *Bifidobacterium longum* BORI for 12 weeks. The gut microbiota was analyzed using 16S rRNA sequencing and bioinformatics. Brain functions were measured using the Consortium to Establish a Registry for Alzheimer’s disease, Satisfaction with life scale, stress questionnaire, Geriatric depression scale, and Positive affect and negative affect schedule. Blood brain-derived neurotrophic factor (BDNF) was determined using enzyme-linked immunosorbent assay. Relative abundance of inflammation-causing gut bacteria was significantly reduced at Week 12 in the probiotics group (*p* < .05). The probiotics group showed greater improvement in mental flexibility test and stress score than the placebo group (*p* < .05). Contrary to placebo, probiotics significantly increased serum BDNF level (*p* < .05). Notably, the gut microbes significantly shifted by probiotics (*Eubacterium* and Clostridiales) showed significant negative correlation with serum BDNF level only in the probiotics group (R_S_ = −0.37, R_S_ = −0.39, *p* < .05). In conclusion, probiotics promote mental flexibility and alleviate stress in healthy older adults, along with causing changes in gut microbiota. These results provide evidence supporting health-promoting properties of probiotics as a part of healthy diet in the older adults.

Aging is characterized by progressive decline in biological functions of the organism (1). The functions of the central nervous system also change during normal aging, leading to age-associated cognitive decline and mood disorders that are common and major health issues among older adults (1). Most industrialized countries are facing a rapid increase in the proportion of older adults considered to be in the danger zone of neurological diseases (1,2). Beyond the increasing risk of health issues, the critical social problems such as high economic burden and low growth potential of an aging society have ensued (2). Therefore, development of efficient preventative and therapeutic strategies targeting neurodegenerative disorders should be considered as a public health priority to promote healthy aging in the global population.

The gut microbiota, a collection of microorganisms found in the gastrointestinal tract, has pivotal roles in anatomical, physiological, and immunological host functions (3,4). The gut microbiota undergoes a significant transition in its composition and function during aging and these alterations can affect health and age-related diseases (5,6). Based on a series of studies, it is now becoming evident that maintaining gut microbial balance during aging is imperative for healthy late life (7). Recently, the emerging concept of gut–brain axis, referring to a bidirectional relationship between gut and brain, has linked gut microbiota to age-related neurodegenerative diseases, such as Alzheimer’s disease, and mood disorders including depression and anxiety (8–12). The interplay between gut and brain involves a complex network of endocrinological, immunological, and neural mediators, which has been considered as a critical target for the manipulation of brain health and neurodegenerative diseases (13–15).

Diet is one of the critical lifestyle factors for physical and mental well-being throughout the life span, including later life (16,17). A growing body of evidence suggests that dietary components or nutrients affect various biological functions including brain activity (10,16,18–20). Therefore, research is actively focusing on the emerging concept of brain health preservation through dietary interventions. Probiotics, as part of a healthy diet, have received increasing attention for their potential to regulate brain health via the microbiota–gut–brain axis (9,21). Probiotic bacteria have been shown to affect intestinal microbial dynamics and homeostasis, and influence the physiology of the intestine and distal organs, including the brain (22). However, most of the current evidence comes from animal experiments, and it is crucial yet challenging to assess whether such findings can be translated to humans. Thus, it is critical to validate the clinical properties and effects of probiotics on human gut and brain health, particularly focusing on independently living older individuals, which can be majorly affected by cognitive and mental disorders. Therefore, we conducted a randomized, double-blind, placebo-controlled, multicenter trial to test our hypothesis that probiotic consumption has beneficial effects on intestinal health, and contributes to ameliorate cognitive and mental impairment in the older adults.

## Materials and Methods

### Study Design

The study was a randomized, double-blind, placebo-controlled, multicenter clinical trial examining the effects of probiotics consumption on intestinal and brain health in elders over the age of 65, conducted at Seoul National University (Seoul, Republic of Korea) and Bundang Jesaeng Hospital (Seongnam, Republic of Korea) from March 2018 to March 2019. The study included a 2-week wash-out period and a 12-week intervention period. During a 2-week wash-out phase, eligible participants were instructed to refrain from dietary supplements including probiotics and other dietary supplements. Participants were then randomly assigned to one of the two following groups: Placebo or Probiotics group. During the intervention period, participants consumed their assigned products twice a day for 12 consecutive weeks. They visited the clinic at baseline (Week 0), Week 4, Week 8, and Week 12 for a compliance check and blood and fecal samples were collected at each visit; and they conducted neuropsychological test at baseline, Week 4 and Week 12. Participants were asked not to change their usual dietary habits and health-related behaviors during the period of intervention. They were asked to record treatment intake, and unusual events such as the use of medication and experiencing adverse events in a daily manner in order to check the adherence to the study. This work is registered with CRiS (Clinical Research Information Service; http://cris.nih.go.kr; https://cris.nih.go.kr/cris/search/search_result_st01_en.jsp?seq=14020&ltype=&rtype=. Registration ID: KCT0003929).

### Participants

Participants were recruited from communities in Seoul and Seongnam in the Republic of Korea. Recruitment flyer was posted at Gwanak-gu Community Health Center (Seoul, Republic of Korea), Seoul W Internal Medicine Clinic (Seoul, Republic of Korea) and Bundang Jesaeng Hospital (Seongnam, Republic of Korea). Candidates were invited to an onsite screening, which includes interviews asking about health history, health-related behavior, and dietary habits. Assessment of physical and cognitive functional status was conducted using activities of daily living, instrumental activities of daily living, and Mini-Mental State Examination (MMSE) by experienced research staff.

#### Criteria for eligibility

Eligible subjects had to be over 65 years old and to consent to be randomly assigned and refrain from consuming any other dietary supplements, which include other probiotics, yogurts with live, active cultures or supplements, and immune-enhancing supplements, during the period of the study. We excluded participants with the use of antibiotics, anti-inflammatory medications, gastrointestinal medicine within the past 3 months; and with regular intake of probiotics within the past 3 months. Participants who are incapable of living independently based on activities of daily living and instrumental activities of daily living score were excluded. A total of 107 candidates entered for screening and a total of 63 subjects enrolled for the study. This study was approved and monitored by the Institutional Review Board of Seoul National University (IRB No. 1801/002-015) and Bundang Jesaeng Hospital (IRB No. IMCN18-01), and written informed consent was obtained from all participants.

### Study Capsules

Participants were provided with either placebo or probiotics. For the probiotics, participants were asked to consume two capsules after the meal in the morning and evening, which made a total of four capsules (a total of 1×109 colony-forming unit of *Bifidobacterium bifidum* BGN4 and *Bifidobacterium longum* BORI in soybean oil) to be taken per day. For the placebo, each capsule contained 500 mg of soybean oil only. Treatment products were not able to distinguish by package, color, taste, and smell in order to maintain treatment allocation concealed from participants and study staff. Test products were provided by Bifido Inc. (Seoul, Republic of Korea).

### Randomization

Study coordinator who was not involved in the study generated a random sequence using GraphPad Prism (version 6.05; GraphPad Software, San Diego, CA) and the random number was stratified by sex with 1:1 allocation. The allocation sequence was concealed from the researchers and details of the allocated group were given on color code containing the sequential number which was prepared by product provider. Independent study coordinator dispensed either placebo or probiotics capsules according to a computer-generated randomized sequence.

### Blinding

All participants, study coordinators, and researchers were blinded throughout the entire study. The study was unblinded after all statistical analyses were completed.

### Sample Collection

Twelve-hour fasting blood samples and stool samples were collected at each visit (baseline, 4th, 8th, and 12th week). Blood samples were collected into serum separating tube and ethylenediaminetetraacetic acid-coated tubes for serum and plasma isolation, respectively. Serum and plasma samples were aliquoted and immediately stored at −80°C for later analysis. For stool sample collection, we provided a stool collection tube that contains DNA stabilizing preservative reagent (Norgen Biotek, Thorold, ON, Canada). We instructed participants, following manufacturer’s instructions, to collect fecal samples into the tubes and mix gently until the stool sample is completely submerged into the preservative. Participants were instructed to collect stool samples within the 48-hour period before visiting; the tubes were kept tightly sealed and stored at room temperature (15–25°C) until they were shipped. After the samples were shipped to the laboratory, aliquots of 180~200 mg of stool samples were immediately stored at −80°C until later analysis.

### Outcome Assessments

The primary outcomes include results from cognitive function and mood tests at the end of the experiment. The secondary outcomes were gut microbial composition and anthropometric assessments measured at each visit; and neuronal biochemistry marker from the blood (brain-derived neurotrophic factor [BDNF]) at the end of the experiment.

#### Anthropometric measures

Body weight and height were measured at each visit using weight scales and stadiometers. BMI was calculated as weight in kilograms divided by height in meters squared.

#### Evaluation of intestinal health

Participants completed a general health questionnaire that asks about improvements in bowel habits at 4th, 8th, and 12th week, respectively. The questionnaire measures 10 bowel habits, asking whether there were improvements in the following parameters in the last 4 weeks: overall bowel health; frequency of defecation; amount of defecation; feeling of incomplete evacuation; stool odor; abdominal cramping; bowel sounds; number of gas passage; abdominal distention; and frequency of diarrhea. Participants responded with a 5-point scale that ranges from 1 (“not at all”) to 5 (“very much”); and the higher scores indicate that there was improvement in each parameter.

#### Gut microbiota analysis

##### Genomic DNA extraction

Total bacterial DNA was isolated from stool by using the QIAamp fast DNA Stool Mini Kit (QIAGEN, Hilden, Germany) according to the manufacturer’s instructions with the following additional steps. Extracted genomic DNA was confirmed via gel electrophoresis and was quantified by spectrophotometer NanoDrop ND-2000 (Thermo Scientific, Waltham, MA).

##### Amplification of 16S rRNA gene and sequencing

Hypervariable regions (V3-V4) of 16S ribosomal ribonucleic acid (rRNA) gene were amplified using barcoded universal primers for each sample. Polymerase chain reaction (PCR) was carried out by using BioFact F-Star taq DNA polymerase (BioFACT, Seoul, Republic of Korea). Briefly, a final volume of 50 μL of PCR reaction contained about 20 ng of DNA template, 5 μL of 10× Taq buffer (20 mM Mg^2+^), 1 μL of 10 mM dNTP mix, 2 μL of forward and reverse barcoded primers (10 pmol/μL), and 0.25 μL of DNA polymerase. PCR reactions were amplified using a GeneAmp PCR system 9700 (Applied Biosystems, Foster City, CA). The PCR program was as follows: initial for 5 minutes hold at 94°C, followed by 28 cycles of denaturation (30 seconds, 95°C), annealing (30 seconds, 60°C), and extension (30 seconds, 72°C), with a final extension step (10 minutes, 72°C) followed by holding at 4°C. The PCR product was confirmed by using 1% agarose gel electrophoresis and visualized under a Gel Doc system (BioRad, Hercules, CA). The amplified products were purified with PureLink Quick Gel Extraction and PCR Purification Combo Kit (Invitrogen, Carlsbad, CA) and quantified by the Qubit 2.0 fluorometer (Invitrogen). The size of library was assessed by BioAnalyzer (Agilent Technologies, Santa Clara, CA). The amplicons were pooled and sequenced with an Illumina MiSeq sequencing system (Illumina, San Diego, CA).

##### Bioinformatic analysis of sequencing data

Microbial sequences were processed using QIIME2 (version 2019.1) (23). Briefly, sequences were denoised to remove the sequences with low-quality score and chimeras via DADA2. Then, denoised sequences were clustered into operational taxonomic units (OTUs) and OTU representative sequences were aligned based on SILVA database (version 132) at 99% sequence identity with scikit-learn Naive Bayes-based machine-learning classifier. A phylogenetic tree was generated using MAFFT and FastTree method for diversity analyses. Downstream analyses on alpha diversity were carried out to measure dissimilarities in richness and evenness of microbial community. Comparisons of relative abundance between groups were performed to identify the differential features across the samples.

#### Evaluation of cognitive function and mood status

The Korean version of the Consortium to Establish a Registry for Alzheimer’s Disease (CERAD-K) was used to measure cognitive function. The CERAD-K, a validated measure for the screening of Alzheimer’s disease, assesses cognitive function including 11 tests measuring domains of language function, memory function, visuo-spatial processing function, and attention and executive function (24).

A validated 20-item self-reported questionnaire was used to ask the level of stress in a category of burn-out, depression, and anger during the past 1 month (25). Participants responded with a 5-point scale that ranges from 1 (“never”) to 5 (“very often”). Total scores were calculated, and higher scores mean higher level of stress. The quality of life (QoL) was measured with the Satisfaction With Life Scale (SWLS), a validated subjected report of global life satisfaction (26). It consists of five items with a 7-point scale that ranges from 1 (“not at all”) to 7 (“very much”). Responses were summed and higher scores indicate higher QoL. The Korean version of Geriatric Depression Scale (GDS-K) was used to evaluate the level of depression. The GDS-K is a 30-item self-reported questionnaire which is a validated instrument for the diagnosis of clinical depression (27). Each question was answered with binary responses (“yes” or “no”) and scored as either 0 or 1 point. The cumulative score is calculated, and the higher score means the higher level of depression. The Positive Affect and Negative Affect Schedule (PANAS) is a validated self-report instrument in the assessment of positive and negative affect (28). The PANAS is comprised of two 10-item scales which measure both positive and negative affect, respectively. Each item is assessed with 5-point scale of 1 (“not at all”) to 5 (“very much”). The summed scores from each positive and negative affect indicate the level positive and negative affect, respectively.

#### Serum biochemical markers

Serum BDNF level was measured using BDNF DuoSet ELISA kit (DY248; R&D Systems, Wiesbaden-Nordenstadt, Germany) and DuoSet Ancillary Reagent kit 2 (DY008; R&D Systems) according to the manufacturer’s instructions. Briefly, sample or standard was added to a plate coated with capture antibody and the plate was incubated for 2 hours at room temperature. After washing the plate sufficiently, detection antibody was added to the plate and the plate was incubated for 2 hours at room temperature. Streptavidin conjugated to horseradish peroxidase was added to each well and the plate was incubated for 20 minutes at room temperature. Then substrate solution was added to each well for 20 minutes of incubation at room temperature following sufficient washing with wash buffer, and the plate was ready for determining the optical density at 450 nm wavelength using a microplate reader (SpectraMax iD3, Molecular Devices, Austria).

### Statistical Analysis

#### Sample size

To detect a significant change in cognitive function with a two-sided 5% significance level and a power of 80%, a sample size of 32 was determined, given a 20% of dropout rate.

#### Analysis plan

The normality assumption and homogeneity of variance were tested by Kolmogrov–Smrinov test for study variables. For the comparison analysis of variables at the baseline between groups, we used independent *t*-test, χ ^2^ tests, or Fisher’s Exact Tests. To compare the difference between groups at each time point and delta value between the visits (Δ(Week 4–Week 0), Δ(Week 8–Week 0), and Δ(Week 12–Week 0)) between the two groups, we performed unpaired *t*-test, Mann–Whitney *U* test or generalized linear model (GLM). To compare the difference between baseline and the data from end point (Week 12), we used paired *t*-test or Wilcoxon signed rank test. To detect the difference between groups over the visits, we used a mixed-model analysis of variance (ANOVA) or Friedman test as a nonparametric alternative to the repeated measures ANOVA. Correlations were assessed by Spearman rank correlation analysis. Correction for multiple testing was performed based on the false discovery rate or Bonferroni correction. The *p* < .05 and false discovery rate <0.05 were considered statistically significant in all statistical analyses. All statistical analyses were conducted using Partek (version 6.6; Partek, Saint Louis, MI), SPSS (version 25.0; SPSS Inc., Chicago, IL), or GraphPad Prism (version 6.0; GraphPad Software, San Diego, CA).

## Results

### General Characteristics of Participants at Baseline

A total of 107 volunteers were screened for eligibility and 63 subjects were enrolled for the study (Supplementary Figure 1). Sixty-three participants were randomized, with 31 and 32 subjects in the placebo and probiotics group, respectively. Fifty-three individuals completed the study and 10 participants withdrew the consent and discontinued the study, and no clinically relevant adverse events were reported during the intervention. When comparing characteristics of participants who withdrew (*N* = 10) and those who completed the trial (*N* = 53), there were no significant differences (Supplementary Table 1). Therefore, we confirmed that randomization was successful. In all analyses, we included data from participants who completed the intervention. Demographic and clinical characteristics at baseline are summarized in Supplementary Table 2. Average age was 72.00 and 71.11 years in the placebo and probiotics group, respectively, with no significant difference (*p* = .4538). The ratio of male to female and BMI did not significantly differ between two groups. Socioeconomic characteristics, including educational level, marital status and type of household, and other health-related characteristics such as cigarette use, alcohol use, physical activity, and self-evaluated health status, were not different between the placebo and probiotics group. In addition, cognitive functions and depression scores, determined by MMSE and Geriatric Depression Scale (GDS-K), were not different between two groups at the baseline.

### Probiotic Supplementation Beneficially Influenced Intestinal Health and Gut Microbial Communities

**Figure 1.**
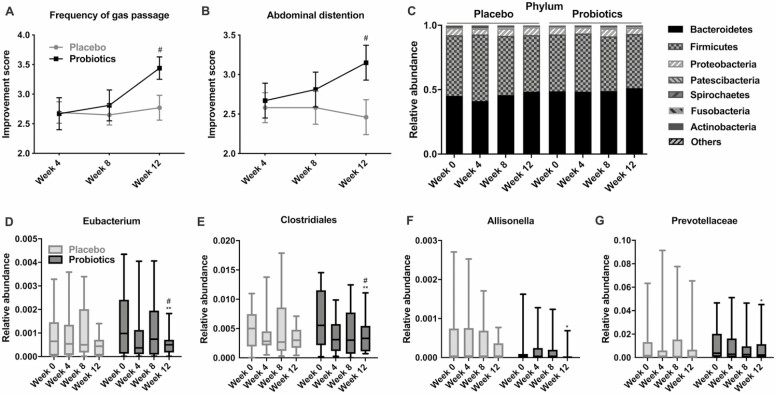
Beneficial influence of probiotic supplementation on intestinal health and gut microbiota. (**A,B**) Improvement scores in frequency of gas passage and abdominal distention measured at each visit are shown. Data are presented as mean (SEM). (**C**) Relative abundance of the gut microbiota at the phylum level and (**D–G**) at the genus level was measured throughout the intervention. Data are presented as mean (min-max). ^#^*p* < .05 based on the Mann–Whitney *U* test; ^**^p < .005, *p < .05 based on a post hoc analysis of Friedman test.

To assess the effect of probiotics on intestinal health, participants filled questionnaire at 4th week, 8th week, and 12th week, respectively, asking whether there were improvements in bowel habits in the last 4 weeks. Bowel habits such as frequency and amount of defecation; feeling of incomplete evacuation; stool odor; number of gas passage; bowel sounds; and abdominal distention were not significantly improved both in the placebo and probiotics group during the intervention period (data not shown); however, scores in frequency of gas passage and abdominal distention showed significant improvements in the probiotics group compared with the placebo (3.44 ± 0.19 vs 2.77 ± 0.21; 3.15 ± 0.22 vs 2.46 ± 0.22, respectively; *p* < .05, Figure 1A and B).

In order to address whether the improvement was driven by any changes in the intestinal bacterial communities, gut microbiome profiling analysis was performed in all participants. Bacterial genomic DNAs from stool samples collected at baseline, 4th week, 8th week, and 12th week were sequenced using 16S rRNA sequencing technology. After preprocessing of bacterial sequences for quality control as described in the Methods, we obtained a total of 10,273,269 raw reads and average reads of 80,260 per sample. To examine the effect of probiotics consumption on gut microbial diversity, we calculated Pielou’s evenness index, Faith’s phylogenetic diversity, observed OTUs, and Shannon’s diversity index. We found no significant changes in the diversity both in the placebo and probiotics group during the intervention period (Supplementary Figure 2A–D). Further, we compared the relative abundance of OTUs and specific bacterial taxa at the different phylogenetic levels. Microbial composition at OTU level showed no significant differences during the intervention both in the placebo and probiotics group (Supplementary Figure 2E). At the phylum level, no significant changes in relative abundance were detected during the intervention both in the placebo and probiotics group (Figure 1C). However, at the genus level, we found significant changes in the gut microbial composition in the probiotics group and no changes in the control group (Figure 1D–G). The relative abundances of *Eubacterium, Allisonella,* Clostridiales, and Prevotellaceae gradually changed during the intervention, and significantly decreased at Week 12 in the probiotics group (*p* < .05).

### Probiotic Supplementation Improved Brain Function and Increased Peripheral BDNF Levels

**Figure 2.**
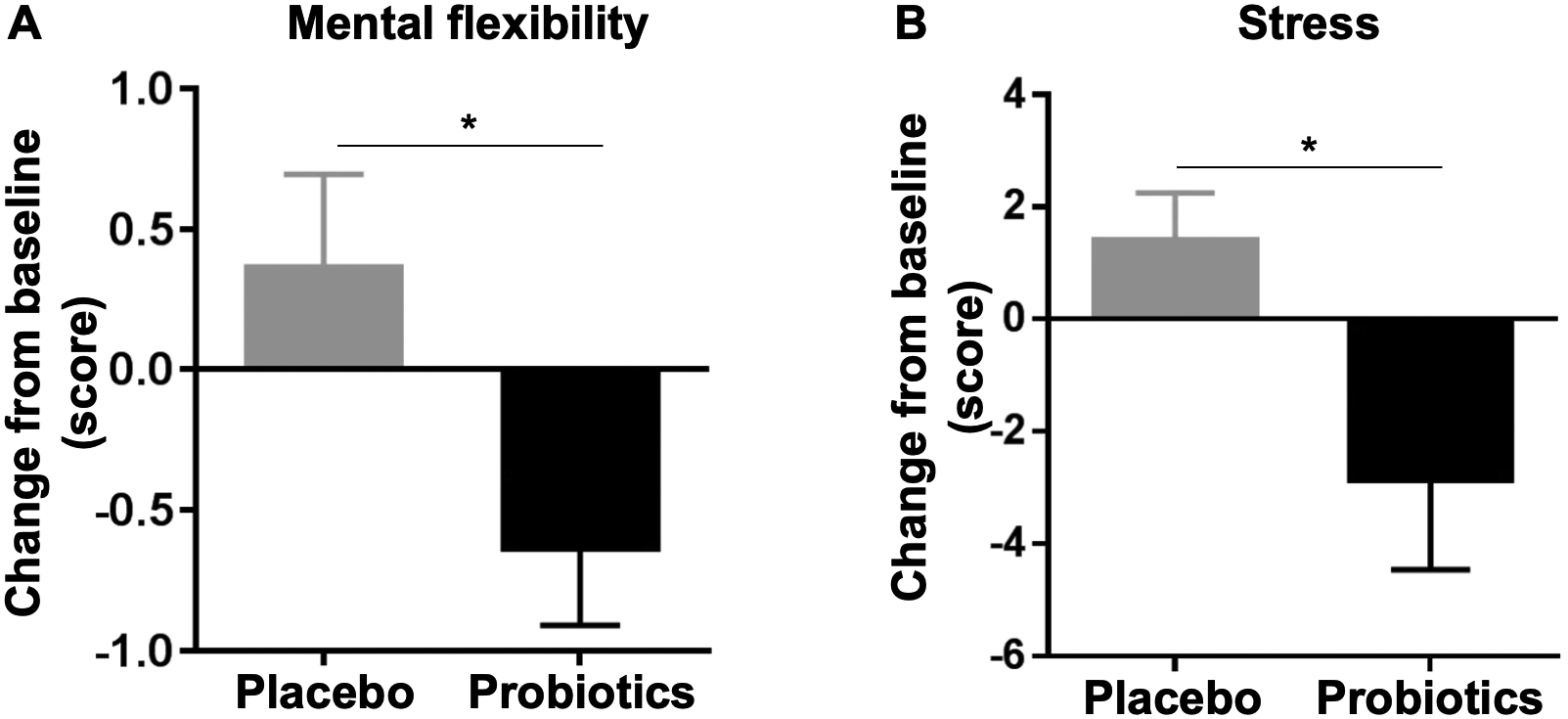
Improved cognitive and mental functioning after probiotic supplementation. (**A**) Change from baseline of cognitive performance score in the mental flexibility test is shown. Reduction in the performance score of mental flexibility indicates improved attention and executive function. (**B**) Change from baseline of stress level is shown. Reduction in the change indicates a reduced level of mental stress. Data are presented as mean (SEM). *p < .05 based on the Mann–Whitney *U* test for the comparison analysis of changes from baseline between the two groups.

To evaluate the impacts of probiotic supplementation on cognitive function, each participant was tested by the CERAD-K, a validated cognitive test battery that scores language, memory, visuo-spatial processing, and attention/executive functions. The assessment was performed at baseline, Week 4, and Week 12 (Table 1). The changes at the fourth week from baseline in the probiotics group were not different from those in the placebo group for all the domains of the cognitive assessment; however, the changes at Week 12 from baseline in the scores of mental flexibility test were significantly different between placebo and probiotics group (Table 2). Interestingly, mental flexibility showed a significant improvement at Week 12 in the probiotics group compared with the placebo group (*p* < .05, Figure 2A). In addition, study subjects filled series of questionnaires to evaluate the impact of probiotics on mood status including quality of life, stress, depression, and positive and negative affect. The 12-week consumption of probiotics did not change the scores of quality of life, GDS-K, and PANAS; however, it did affect the stress score (Table 2). While the stress score was increased in the placebo group (1.38 ± 0.86), it was dramatically decreased in the probiotics group (−2.85 ± 1.16; *p* < .05, Figure 2B).

**Table 1.** Cognitive Function Before and After the Intervention

	Placebo (*N* = 26)			^a^ *p*-value	Probiotics (*N* = 27)			^a^ *p*-value	^b^ *p* for Δ(Week 4–Week 0)	^b^ *p* for Δ(Week 12–Week 0)
	Week 0	Week 4	Week 12		Week 0	Week 4	Week 12			
Language function										
Verbal fluency	14.96 (4.05)	16.42 (4.59)*	16.88 (4.55)	.01	14.44 (4.48)	15.67 (5.17)	15.41 (4.17)	.40	.86	.39
Boston naming test	11.69 (2.19)	12.19 (2.26)	12.23 (2.23)	.28	12.15 (1.56)	12.70 1.49)	12.96 (1.34)^**^	<.005	.70	.23
Memory function										
Word list encoding	18.92 (4.42)	20.77 (3.63)	22.23 (4.74)^**^	<.001	18.26 (2.81)	21.33 (3.10)^**^	22.22 (3.79)^**^	<.001	.12	.47
Word list recall	6.38 (1.92)	7.27 (1.78)	7.54 (1.92)*	.01	6.19 (1.82)	6.85 (1.88)	7.52 (1.65)^**^	<.005	.58	.68
Word list savings	83.83 (18.87)	94.24 (14.47)	92.21 (16.50)	.13	84.68 (23.06)	83.44 (19.30)^†^	89.30 (13.27)	.18	.15	.71
Word list recognition	8.77 (1.77)	9.27 (1.25)	9.23 (1.53)	.09	9.22 (0.97)	9.37 (0.74)	9.63 (0.63)	.30	.38	.88
Constructional praxis recall	8.15 (2.81)	8.88 (2.57)	9.23 (2.30)	.02	7.93 (3.05)	8.89 (2.39)	9.52 (1.72)*	<.001	.90	.42
Visuo-spatial processing function										
Constructional praxis	10.04 (1.43)	10.00 (1.60)	10.27 (1.34)	.36	10.00 (1.64)	10.33 (1.44)	10.52 (1.01)	.31	.55	.74
Attention and executive function										
Trail making test A	61.88 (26.82)	49.73 (17.99)*	47.35 (15.91)^**^	<.001	47.33 (18.78)^†^	46.22 (21.47)	44.41 (21.62)	.06	.36	.21
Trail making test B	189.69 (82.28)	164.65 (77.22)	161.19 (78.26)	.15	172.59 (86.95)	148.26 (67.32)	131.11 (58.44)*	.01	.97	.39
Mental flexibility	2.15 (0.97)	2.46 (1.54)	2.52 (1.68)	.48	2.72 (1.56)	2.30 (0.96)	2.08 (0.85)	.10	.06	.03
Digit span test	13.35 (5.18)	14.23 (4.23)	13.65 (4.54)	.59	13.41 (4.48)	15.33 (3.81)^**^	14.59 (3.88)*	<.005	.23	.16

*Note*: Data are presented as mean (*SD*).

^a^ *p*-value from Friedman test; ^b^*p*-value from the Mann–Whitney *U* test or GLM analysis (after adjusting for baseline value) for between-group analysis for the comparison of delta value; ^**^p < .005, *p < .05 based on a post hoc analysis of Friedman test; ^†^*p* < .05 based on the Mann–Whitney *U* test for between-group analysis at each time point.

**Table 2.** Mood Status Before and After the Intervention

	Placebo (*N* = 26)			^a^ *p*-value	Probiotics (*N* = 27)			^a^ *p*-value	^b^ *p* for Δ(Week 4– Week 0)	^b^ *p* for Δ(Week 1 2–Week 0)
	Week 0	Week 4	Week 12		Week 0	Week 4	Week 12			
QoL	22.88 (7.26)	21.38 (5.45)	21.58 (6.68)	.07	20.74 (5.85)	20.33 (6.77)	20.89 (6.93)	.38	.12	.18
Stress	31.12 (12.24)	31.73 (13.17)	32.50 (11.46)	.37	31.74 (11.77)	29.33 (8.61)	28.89 (7.77)	.10	.10	.04
Depression	6.31 (5.87)	6.23 (6.16)	6.15 (5.72)	.74	7.59 (4.61)	6.96 (5.36)	6.41 (5.35)	.12	.27	.39
Positive affect	28.46 (7.89)	26.08 (7.20)*	25.58 (6.25)*	.09	28.59 (6.69)	27.52 (6.35)	28.41 (8.28)	.86	.59	.12
Negative affect	16.62 (6.54)	15.54 (5.41)	15.54 (5.27)	.39	14.78 (4.73)	14.67 (4.54)	14.04 (4.75)	.66	.88	.69

*Note*: Data are presented as mean (*SD*). QoL = Quality of life.

^a^ *p*-value from Friedman test; ^b^*p*-value from the Mann–Whitney *U* test for between-group analysis for the comparison of delta; ^**^p < .005, *p < .05 based on a post hoc analysis of Friedman test; ^†^*p* < .05 based on the Mann–Whitney *U* test for between-group analysis at each time point.

**Figure 3.**
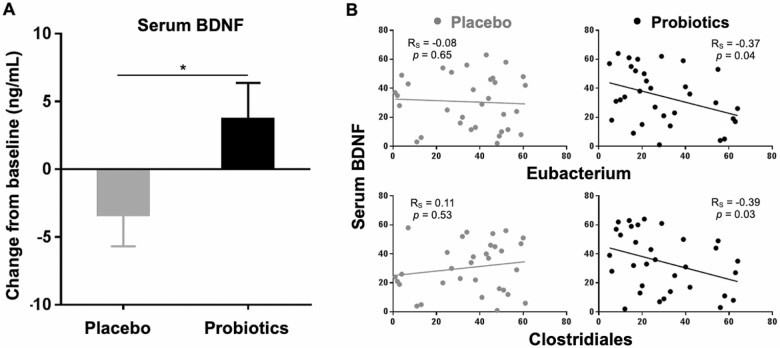
Elevated level of serum BDNF after probiotic supplementation. (**A**) Change from baseline of serum BDNF is shown. Data are presented as mean (SEM). *p < .05 for time × treatment from a mixed-model analysis of variance. (**B**) Scatter diagrams with regression lines show the relationship between relative abundance of shifted gut bacteria after probiotic supplementation and the level of serum BDNF. Measurements were rank-normalized and plotted separately for the placebo and probiotics group. Correlation coefficient (R_S_) and *p*-values based on Spearman rank correlation analysis. BDNF = Brain-derived neurotrophic factor.

The observations that probiotic supplementation improved the cognitive function and mental stress prompted us to determine the level of BDNF in blood. BDNF is a neurotrophic factor known to be crucial for learning, memory function, and stress. In contrast to the placebo group (−3.32 ± 2.35), serum BDNF level was significantly increased at Week 12 in the probiotics group (3.68 ± 2.69; *p* < .05, Figure 3A). In addition, to address the question of whether changes in intestinal bacterial communities be related to the serum level of BDNF, we conducted correlation analysis between the relative abundance of each genera and the level of BDNF. It is of interest that *Eubacterium* and Clostridiales showed a significant negative correlation with the level of serum BDNF only in the probiotics group (R_S_ = −0.37 and R_S_ = −0.39; *p* < .05, Figure 3B). These findings suggest that reduction in the relative abundances of *Eubacterium* and Clostridiales in the gut driven by probiotic supplementation closely related to the increase in the serum BDNF, thereby improving brain functions.

## Discussion

In the present study, we conducted a randomized, double-blind, placebo-controlled, multicenter trial to address the impact of probiotics on intestinal health and how they contribute to ameliorating cognitive and mental decline in the older adults. Our findings demonstrate that probiotics have system-wide effects on the gut–brain axis in healthy community-dwelling older adults by promoting cognitive and mental health and changing the gut microbial composition.

Emerging evidence has suggested that probiotics have considerable impacts on various cerebral functions through the regulation of the gut–brain axis, but the current studies are mainly focused on patients with mild cognitive impairment, Alzheimer’s disease, and major depressive disorder (29–32). However, while neurodegenerative disorders and psychological distress are a common threat to well-being in old age, nutritional intervention to prevent or delay age-associated decline in brain function in the general older population is still underexplored. In fact, there is only one report on the effects of probiotic consumption in healthy older adults, showing that milk fermented by *Lactobacillus helveticus* IDCC3801 improved cognitive functions in healthy older adults (33). However, the sample size was too small and the criteria for the study participants did not represent the general population of older adults. Therefore, the critical need for clinical studies in the general population has been raised. To the best of our knowledge, this is the first well-controlled clinical study demonstrating system-wide effects of probiotics on the gut–brain axis, which encompasses the large-scale analysis of the gut microbiota and multiple aspects of brain functions in healthy older population.

Randomized controlled trials (RCTs) are very challenging for several issues but the most rigorous method, which provides the most reliable evidence for clinical practice; however, there are few RCTs specifically designed for older adults because it is difficult to recruit older people, particularly community-dwelling older adults (34–36). Therefore, older adults have been excluded from clinical trials and most studies focused on older group of patients (36). In addition, it is relatively hard to follow up and contact older people during a trial which increases the risk of dropout and reduces compliance (36,37). Despite these challenges, in the present study, participant compliance was good as the average rate of compliance to intervention was 96.5%, with a dropout rate of 15.9% only. Moreover, it is important to note that the present study recruited older adults without diseases, not focusing on good responders to a treatment effect such as patients with neurological disorders, which makes our findings more applicable as a generalized health care strategy in community-dwelling older population.

In the probiotics group, the gut microbial composition shifted gradually, and the most relevant change was the reduction in the abundance of bacteria that cause inflammation including *Eubacterium*, *Allisonella*, and Prevotellaceae. It has been identified that *Eubacterium* and Prevotellaceae species, which were significantly reduced after probiotic consumption, are proinflammatory microbiota associated with autoimmune disease and chronic intestinal inflammation in mice (38,39). Of note, the genus *Allisonella*, whose abundance was significantly reduced in the probiotics group, produces histamine, a biogenic monoamine inducing proinflammatory response both centrally and systemically (40). Moreover, in patients with Alzheimer’s disease, elevated levels of histamine stimulate neuroinflammation via induction of low-grade systemic inflammation (40). Therefore, these findings may parallel our hypothesis that probiotic supplementation in the older adults may negatively affect inflammaging, a characteristic of chronic low-grade inflammatory status in older adults, via the modulation of microbial composition. However, further studies are required to assess whether the probiotic supplementation affects immunological mechanisms.

The findings of the present study suggest that interaction between the gut microbiota and the central nervous system may underlie the improvements in cognitive and cerebral functioning upon probiotic supplementation and explain the concomitant changes in peripheral neuromodulators. BDNF, a neurotrophic factor vital for synaptic formation, plasticity, and neuroimmune responses, has long been studied to assess its critical role in learning, memory formation, and affective disorders (41,42). Previously, the influence of diet and nutrition on BDNF has been explored; and serum BDNF has been shown to be increased in response to dietary supplements in humans. For example, a 1-week of oral consumption of α-linolenic acid increased the level of serum BDNF in healthy young adults (43). Also, a 6-week supplementation with natural extracts rich in flavonoids and polyphenolic compounds enhanced serum BDNF levels in physically active men (44). In the present study, it was notable that the beneficial impact of a 12-week probiotic intervention on serum BDNF levels was evident in older population. More recently, BDNF has emerged as a pivotal link in the gut–brain axis (41,42). Several studies demonstrated that gut dysbiosis correlates with reduced expression of BDNF, which alters cognitive function and triggers anxiety-like behavior in germ-free animals (45,46), supporting a role of BDNF in the gut–brain axis. Interestingly, we observed that the relative abundance of significantly shifted gut microbes correlated with the level of serum BDNF in the probiotics group only. This indicates that administration of probiotics may affect the interaction between the gut microbiome and the host BDNF, thereby improving brain functions. Overall, the evidence from this study shows that the shifts in microbial community mirrored changes in the cognitive and mental scores.

Several mechanisms could explain the interaction between changes in abundance of commensal bacteria and brain function observed in the probiotics group. First, it is plausible that the production of neurotransmitters, such as γ-Aminobutyric acid (GABA), dopamine, acetylcholine, serotonin, by commensal bacteria, and neurochemicals including BDNF, may directly or indirectly modulate cognition and mood status (47). As shown in a previous study, probiotic administration influences GABA receptor throughout the brain, with reduced stress-induced anxiety- and depression-like behaviors in rodents (48). Moreover, inflammation-mediated pathways might initiate the pathogenesis of neurodegeneration via the microbiota–gut–brain axis. With respect to inflammaging during normal aging, chronic low-grade inflammation in older adults may affect neuroinflammation by modulating glial cells, which stimulates cognitive impairment (47). One of the routes to translate systemic inflammatory signals into the brain is stimulation of microglia by peripheral cytokines that cross the blood–brain barrier, leading to a proinflammatory status in the brain and dysregulation of neurological processes (47). Additionally, the immunomodulatory roles of circulating immune cells in neuroplasticity also affect the expression of BDNF (49,50). Therefore, it is plausible that mitigation of inflammaging in older adults with probiotic intervention might positively impact on cognitive and mental functions via the modulation of BDNF signaling. Further studies are needed to clearly demonstrate the effect of probiotics on inflammatory status and gut microbiome at the functional level.

The present study is not without limitations. First, direct evidence of improvement in peripheral and cerebral inflammation by probiotic consumption is lacking, which might be the crucial interface linking the gut–brain axis in the present study. Therefore, further mechanistic studies might be needed to elucidate the role of probiotic supplementation by finding biomarkers to link the axis. Second, although cognitive functions were evaluated by validated neuropsychological assessment battery tasks taking at least 60 minutes per subject by a professionally trained panel, psychological assessments of mood status were based on the participants’ self-reporting; therefore, possible recall bias may exist. Additionally, although our results indicated that the benefit of 3 months duration of probiotic intervention was evident, there were no significant changes in some of the cognitive functions in the neuropsychological assessment battery in which we assume that the study duration was not enough to monitor the improvements. Therefore, further studies are required with a longer period of intervention. Despite these limitations, this is the first study examining the effects of probiotic supplementation on brain functions in community-dwelling older adults. In conclusion, our study showed that probiotic supplementation is beneficial for improving cognitive and mental health in community-dwelling healthy older adults with changes in gut microbial composition. These results provide evidence that probiotics have health-promoting properties as part of a healthy diet in the general population of independently living older adults.

## Supplementary Material

glaa090_suppl_Supplementary_Figure

glaa090_suppl_Supplementary_Material

## References

[1] Mattson MP , Arumugam TV. Hallmarks of brain aging: adaptive and pathological modification by metabolic states. Cell Metab. 2018;27:1176–1199. doi: 10.1016/j.cmet.2018.05.011 29874566 PMC6039826

[2] World Health Organization. World Report on Ageing and Health. Geneva: World Health Organization; 2015.

[3] Guarner F , Malagelada JR. Gut flora in health and disease. Lancet. 2003;361:512–519. doi: 10.1016/S0140-6736(03)12489-0 12583961

[4] Rooks MG , Garrett WS. Gut microbiota, metabolites and host immunity. Nat Rev Immunol. 2016;16:341–352. doi: 10.1038/nri.2016.42 27231050 PMC5541232

[5] O’Toole PW , Jeffery IB. Gut microbiota and aging. Science. 2015;350:1214–1215. doi: 10.1126/science.aac8469 26785481

[6] Vaiserman AM , Koliada AK, Marotta F. Gut microbiota: a player in aging and a target for anti-aging intervention. Ageing Res Rev. 2017;35:36–45. doi: 10.1016/j.arr.2017.01.001 28109835

[7] Clark RI , Walker DW. Role of gut microbiota in aging-related health decline: insights from invertebrate models. Cell Mol Life Sci. 2018;75:93–101. doi: 10.1007/s00018-017-2671-1 29026921 PMC5754256

[8] Grenham S , Clarke G, Cryan JF, Dinan TG. Brain-gut-microbe communication in health and disease. Front Physiol. 2011;2:94. doi: 10.3389/fphys.2011.00094 22162969 PMC3232439

[9] Sharon G , Sampson TR, Geschwind DH, Mazmanian SK. The central nervous system and the gut microbiome. Cell. 2016;167:915–932. doi: 10.1016/j.cell.2016.10.027 27814521 PMC5127403

[10] Sandhu KV , Sherwin E, Schellekens H, Stanton C, Dinan TG, Cryan JF. Feeding the microbiota-gut-brain axis: diet, microbiome, and neuropsychiatry. Transl Res. 2017;179:223–244. doi: 10.1016/j.trsl.2016.10.002 27832936

[11] Foster JA , McVey Neufeld KA. Gut-brain axis: how the microbiome influences anxiety and depression. Trends Neurosci. 2013;36:305–312. doi: 10.1016/j.tins.2013.01.005 23384445

[12] Seo D-O , Holtzman DM. Gut microbiota: from the forgotten organ to a potential key player in the pathology of Alzheimer’s disease. J Gerontol A Biol Sci Med Sci. 2019;18:glz262. doi:10.1093/gerona/glz262 PMC7302187 31738402

[13] Forsythe P , Bienenstock J, Kunze WA. Vagal pathways for microbiome-brain-gut axis communication. Adv Exp Med Biol. 2014;817:115–133. doi: 10.1007/978-1-4939-0897-4_5 24997031

[14] Dinan TG , Cryan JF. The microbiome-gut-brain axis in health and disease. Gastroenterol Clin North Am. 2017;46:77–89. doi: 10.1016/j.gtc.2016.09.007 28164854

[15] Sun Y , Baptista LC, Roberts LM, et al The gut microbiome as a therapeutic target for cognitive impairment. J Gerontol A Biol Sci Med Sci. 2019. doi:10.1093/gerona/glz281 PMC7302188 31811292

[16] Moore K , Hughes CF, Ward M, Hoey L, McNulty H. Diet, nutrition and the ageing brain: current evidence and new directions. Proc Nutr Soc. 2018;77:152–163. doi: 10.1017/S0029665117004177 29316987

[17] Grønning K , Espnes GA, Nguyen C, et al. Psychological distress in elderly people is associated with diet, wellbeing, health status, social support and physical functioning- a HUNT3 study. BMC Geriatr. 2018;18:205. doi: 10.1186/s12877-018-0891-3 30180808 PMC6123986

[18] O’Neil A , Quirk SE, Housden S, et al. Relationship between diet and mental health in children and adolescents: a systematic review. Am J Public Health. 2014;104:e31–e42. doi: 10.2105/AJPH.2014.302110 PMC4167107 25208008

[19] Oddy WH , Allen KL, Trapp GSA, et al. Dietary patterns, body mass index and inflammation: pathways to depression and mental health problems in adolescents. Brain Behav Immun. 2018;69:428–439. doi: 10.1016/j.bbi.2018.01.002 29339318

[20] Opie RS , Itsiopoulos C, Parletta N, et al. Dietary recommendations for the prevention of depression. Nutr Neurosci. 2017;20:161–171. doi: 10.1179/1476830515Y.0000000043 26317148

[21] Kim CS , Shin DM. Probiotic food consumption is associated with lower severity and prevalence of depression: a nationwide cross-sectional study. Nutrition. 2019;63-64:169–174. doi: 10.1016/j.nut.2019.02.007 31029044

[22] Blander JM , Longman RS, Iliev ID, Sonnenberg GF, Artis D. Regulation of inflammation by microbiota interactions with the host. Nat Immunol. 2017;18:851–860. doi: 10.1038/ni.3780 28722709 PMC5800875

[23] Caporaso JG , Kuczynski J, Stombaugh J, et al. QIIME allows analysis of high-throughput community sequencing data. Nat Methods. 2010;7:335–336. doi: 10.1038/nmeth.f.303 20383131 PMC3156573

[24] Lee JH , Lee KU, Lee DY, et al Development of the Korean Version of the Consortium to Establish a Registry for Alzheimer’s Disease Assessment Packet (CERAD-K) clinical and neuropsychological assessment batteries. J Gerontol B Psychol Sci Soc Sci. 2002;57:P47–P53. doi:10.1093/geronb/57.1.p47 11773223

[25] Lee E , Shin H, Yang Y, Cho J, Ahn K, Kim S. Development of the stress questionnaire for KNHANES: report of scientific study service. Seoul: Korea Centers for Disease Control and Prevention; 2010.

[26] Diener E , Emmons RA, Larsen RJ, Griffin S. The satisfaction with life scale. J Pers Assess. 1985;49:71–75. doi: 10.1207/s15327752jpa4901_13 16367493

[27] Bae JN , Cho MJ. Development of the Korean version of the Geriatric Depression Scale and its short form among elderly psychiatric patients. J Psychosom Res. 2004;57:297–305. doi: 10.1016/j.jpsychores.2004.01.004 15507257

[28] Lee H-H , Kim E-J, Lee M-K. A validation study of Korea positive and negative affect schedule: the PANAS scales. Korean J Clin Psychol. 2003;22:935–946.

[29] Akbari E , Asemi Z, Daneshvar Kakhaki R, et al. Effect of probiotic supplementation on cognitive function and metabolic status in Alzheimer’s disease: a randomized, double-blind and controlled trial. Front Aging Neurosci. 2016;8:256. doi: 10.3389/fnagi.2016.00256 27891089 PMC5105117

[30] Rudzki L , Ostrowska L, Pawlak D, et al. Probiotic *Lactobacillus plantarum* 299v decreases kynurenine concentration and improves cognitive functions in patients with major depression: a double-blind, randomized, placebo controlled study. Psychoneuroendocrinology. 2019;100:213–222. doi: 10.1016/j.psyneuen.2018.10.010 30388595

[31] Kazemi A , Noorbala AA, Azam K, Eskandari MH, Djafarian K. Effect of probiotic and prebiotic vs placebo on psychological outcomes in patients with major depressive disorder: a randomized clinical trial. Clin Nutr. 2019;38:522–528. doi: 10.1016/j.clnu.2018.04.010 29731182

[32] Akkasheh G , Kashani-Poor Z, Tajabadi-Ebrahimi M, et al. Clinical and metabolic response to probiotic administration in patients with major depressive disorder: a randomized, double-blind, placebo-controlled trial. Nutrition. 2016;32:315–320. doi: 10.1016/j.nut.2015.09.003 26706022

[33] Chung Y-C , Jin H-M, Cui Y, et al Fermented milk of *Lactobacillus helveticus* IDCC3801 improves cognitive functioning during cognitive fatigue tests in healthy older adults. J. Funct. Foods. 2014;10:465–474. doi: 10.1016/j.jff.2014.07.007

[34] Kammerer K , Falk K, Herzog A, Fuchs J. How to reach ‘hard-to-reach’older people for research: the TIBaR model of recruitment. Survey Methods: Insights from the Field (SMIF). 2019. Retrieved from https://surveyinsights.org/?p=11822. doi: 10.25646/6315

[35] Broekhuizen K , Pothof A, de Craen AJ, Mooijaart SP. Characteristics of randomized controlled trials designed for elderly: a systematic review. PLoS One. 2015;10:e0126709. doi: 10.1371/journal.pone.0126709 25978312 PMC4433127

[36] Clegg A , Relton C, Young J, Witham M. Improving recruitment of older people to clinical trials: use of the cohort multiple randomised controlled trial design. Age Ageing. 2015;44:547–550. doi: 10.1093/ageing/afv044 25857552

[37] Ungar A , Marchionni N. Cardiac Management in the Frail Elderly Patient and the Oldest Old. Cham: Springer; 2017.

[38] Palm NW , de Zoete MR, Cullen TW, et al. Immunoglobulin A coating identifies colitogenic bacteria in inflammatory bowel disease. Cell. 2014;158:1000–1010. doi: 10.1016/j.cell.2014.08.006 25171403 PMC4174347

[39] Zhou C , Zhao H, Xiao X-y, et al Metagenomic profiling of the pro-inflammatory gut microbiota in ankylosing spondylitis. J Autoimmun. 2020;107:102360. 31806420 10.1016/j.jaut.2019.102360

[40] Westfall S , Lomis N, Kahouli I, Dia SY, Singh SP, Prakash S. Microbiome, probiotics and neurodegenerative diseases: deciphering the gut brain axis. Cell Mol Life Sci. 2017;74:3769–3787. doi: 10.1007/s00018-017-2550-9 28643167 PMC11107790

[41] Licinio J , Wong M-. Brain-derived neurotrophic factor (BDNF) in stress and affective disorders. Mol Psychiatry. 2002;7:519. doi: 10.1038/sj.mp.4001211 12140770

[42] Bauer KC , Huus KE, Finlay BB. Microbes and the mind: emerging hallmarks of the gut microbiota-brain axis. Cell Microbiol. 2016;18:632–644. doi: 10.1111/cmi.12585 26918908

[43] Hadjighassem M , Kamalidehghan B, Shekarriz N, et al. Oral consumption of α-linolenic acid increases serum BDNF levels in healthy adult humans. Nutr J. 2015;14:20. doi: 10.1186/s12937-015-0012-5 25889793 PMC4353682

[44] Sadowska-Krępa E , Kłapcińska B, Pokora I, Domaszewski P, Kempa K, Podgórski T. Effects of six-week Ginkgo biloba supplementation on aerobic performance, blood pro/antioxidant balance, and serum brain-derived neurotrophic factor in physically active men. Nutrients. 2017;9:803. doi: 10.3390/nu9080803 28933745 PMC5579597

[45] Diaz Heijtz R , Wang S, Anuar F, et al. Normal gut microbiota modulates brain development and behavior. Proc Natl Acad Sci USA 2011;108:3047–3052. doi: 10.1073/pnas.1010529108 21282636 PMC3041077

[46] Gareau MG , Wine E, Rodrigues DM, et al. Bacterial infection causes stress-induced memory dysfunction in mice. Gut. 2011;60:307–317. doi: 10.1136/gut.2009.202515 20966022

[47] Di Benedetto S , Müller L, Wenger E, Düzel S, Pawelec G. Contribution of neuroinflammation and immunity to brain aging and the mitigating effects of physical and cognitive interventions. Neurosci Biobehav Rev. 2017;75:114–128. doi: 10.1016/j.neubiorev.2017.01.044 28161508

[48] Bravo JA , Forsythe P, Chew MV, et al. Ingestion of Lactobacillus strain regulates emotional behavior and central GABA receptor expression in a mouse via the vagus nerve. Proc Natl Acad Sci USA 2011;108:16050–16055. doi: 10.1073/pnas.1102999108 21876150 PMC3179073

[49] Gibney SM , McGuinness B, Prendergast C, Harkin A, Connor TJ. Poly I:C-induced activation of the immune response is accompanied by depression and anxiety-like behaviours, kynurenine pathway activation and reduced BDNF expression. Brain Behav Immun. 2013;28:170–181. doi: 10.1016/j.bbi.2012.11.010 23201589

[50] Cortese GP , Barrientos RM, Maier SF, Patterson SL. Aging and a peripheral immune challenge interact to reduce mature brain-derived neurotrophic factor and activation of TrkB, PLCγ1, and ERK in hippocampal synaptoneurosomes. J Neurosci. 2011;31:4274–4279. doi: 10.1523/JNEUROSCI.5818-10.2011 21411668 PMC3086395

