# Supplementary figures and images for: Probiotic Supplementation Improves Cognitive Function and Mood with Changes in Gut Microbiota in Community-Dwelling Older Adults: A Randomized, Double-Blind, Placebo-Controlled, Multicenter Trial
Source: J Gerontol A Biol Sci Med Sci. 2020 Apr 17;76(1):32–40. doi: 10.1093/gerona/glaa090 (PMC7861012; doi:10.1093/gerona/glaa090)

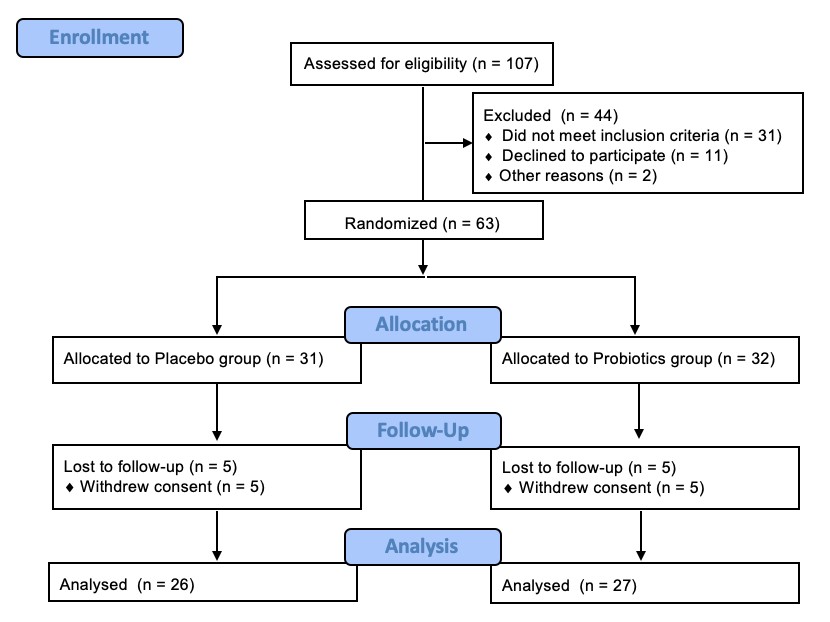

Supplement: glaa090_suppl_Supplementary_Figure [file glaa090_suppl_supplementary_figure.jpeg]
