# Supplementary material for: Probiotic Supplementation Improves Cognitive Function and Mood with Changes in Gut Microbiota in Community-Dwelling Older Adults: A Randomized, Double-Blind, Placebo-Controlled, Multicenter Trial
Source: J Gerontol A Biol Sci Med Sci. 2020 Apr 17;76(1):32–40. doi: 10.1093/gerona/glaa090 (PMC7861012; doi:10.1093/gerona/glaa090)
Supplement: glaa090_suppl_Supplementary_Material [file glaa090_suppl_supplementary_material.docx]

**Supplemental Material**

**Supplemental table 1. General characteristics of participants who withdrew and those who completed the trial**

|  | **Dropout (N = 10)** | **Completer (N = 53)** | ***p*- value** |
| --- | --- | --- | --- |
| **Age, *yrs*** | 75.78 (6.55) | 71.55 (4.30) | 0.08 |
| **Sex** |  |  |  |
| Male | 5 (50%) | 26 (49.06%) | 0.73 |
| Female | 5 (50%) | 27 (50.94%) |  |
| **BMI, *kg/m^2^*** | 22.52 (3.23) | 23.94 (2.84) | 0.22 |
| **Smoking status** | | | |
| Never | 6 (60%) | 42 (79.24%) | 0.17 |
| Ever | 3 (30%) | 5 (9.43%) |  |
| Current | 1 (10%) | 5 (9.43%) |  |
| **Drinking status** | | | |
| Never | 3 (30%) | 24 (45.28%) | 0.80 |
| Ever | 3 (30%) | 5 (9.44%) |  |
| Current | 4 (60%) | 24 (45.28%) |  |
| **Physical activity** | | | |
| No | 1 (10%) | 3 (5.66%) | 0.13 |
| Yes | 9 (90%) | 50 (94.34%) |  |
| **Health status** | | | |
| Excellent | 2 (20%) | 13 (24.53%) | 0.78 |
| Good | 4 (40%) | 33 (62.26%) |  |
| Fair | 3 (30%) | 6 (11.32%) |  |
| Poor | 1 (10%) | 0 (0%) |  |
| Very poor | 0 (0%) | 1 (1.89%) |  |
| **MMSE score** | 25.00 (2.78) | 26.77 (2.33) | 0.08 |
| **GDS-K score** | 6.50 (5.50) | 6.96 (5.24) | 0.81 |

*Note*. Data are presented as mean (SD) or N (%). P-value based on t-test, chi-squared test, or Fisher’s exact test. Abbreviations: GDS-K, The Korean version of Geriatric depression scale; MMSE, Mini mental state examination.

**Supplemental table 2. General characteristics of participants at baseline**

|  | **Placebo (N = 26)** | **Probiotics (N = 27)** | ***p*- value** |
| --- | --- | --- | --- |
| **Age, *yrs*** | 72.00 (3.36) | 71.11 (5.02) | 0.4538 |
| **Sex** |  |  |  |
| Male | 16 (61.54) | 10 (37.04) | 0.0700 |
| Female | 10 (38.46) | 17 (62.96) |  |
| **BMI, *kg/m^2^*** | 24.72 (02.93) | 23.19 (2.63) | 0.0511 |
| **Education** |  |  |  |
| Elementary or less | 3 (11.54) | 6 (22.22) | 0.5694 |
| Junior-high school | 6 (23.08) | 6 (22.22) |  |
| High school | 6 (23.08) | 8 (29.63) |  |
| College or more | 11 (42.30) | 7 (25.93) |  |
| **Marital status** |  |  |  |
| Divorced/separated | 1 (3.85) | 3 (11.11) | 0.1659 |
| Widowed | 1 (3.85) | 5 (18.52) |  |
| Married | 24 (92.30) | 19 (70.37) |  |
| **Lives** |  |  |  |
| Alone | 2 (7.69) | 6 (22.22) | 0.2501 |
| With others | 24 (92.31) | 21 (77.77) |  |
| **Smoking status** |  |  |  |
| Never | 18 (69.23) | 24 (89.89) | 0.0555 |
| Ever | 3 (12.54) | 3 (11.11) |  |
| Current | 5 (19.23) | 0 (0) |  |
| **Drinking status** |  |  |  |
| Never | 13 (50.00) | 11 (41.74) | 0.4786 |
| Ever | 1 (3.85) | 4 (14.81) |  |
| Current | 12 (46.15) | 12 (44.44) |  |
| **Physical activity** |  |  |  |
| No | 1 (3.85) | 2 (7.41) | 0.5144 |
| Yes | 25 (96.15) | 25 (92.59) |  |
| **Health status** |  |  |  |
| Excellent | 3 (11.54) | 10 (37.04) | 0.0747 |
| Good | 19 (73.08) | 14 (51.85) |  |
| Fair | 4 (15.38) | 2 (7.41) |  |
| Poor | 0 (0) | 0 (0) |  |
| Very poor | 0 (0) | 1 (3.70) |  |
| **Energy intake, *kcal/d*** | 1589.99 (367.75) | 1517.22 (422.86) | 0.5229 |
| **MMSE score** | 26.62 (2.25) | 26.93 (2.42) | 0.6304 |
| **GDS-K score** | 5.04 (4.78) | 6.30 (4.94) | 0.3511 |

*Note*. Data are presented as mean (SD) or N (%). P-value based on t-test, chi-squared test, or Fisher’s exact test. Abbreviations: GDS-K, The Korean version of Geriatric depression scale; MMSE, Mini mental state examination.

**Supplemental figure 1. CONSORT flow diagram**

CONSORT flow diagram of the process through the phases of the randomized controlled trial

**Supplemental figure 2. Diversity and OTU abundance analyses of gut microbiota**

Gut microbial diversity indices: (A) Pielou’s evenness index, (B) Faith’s phylogenetic diversity, (C) observed OTUs, and (D) Shannon’s diversity index, were calculated. Data are presented as mean (min-max). (E) Relative abundance levels of individual OTUs are visualized as a heatmap and clustered using hierarchical clustering analysis. Heatmap is in log 2 normalized relative abundances. Red and blue color in the heatmap indicate OTUs with high and low abundances, respectively. Abbreviations: OTU, Operational taxonomic unit.
